# Intraoperative extracorporeal support for lung transplant: a systematic review and network meta-analysis

**DOI:** 10.1186/s44158-024-00214-x

**Published:** 2024-12-18

**Authors:** Tommaso Pettenuzzo, Honoria Ocagli, Nicolò Sella, Alessandro De Cassai, Francesco Zarantonello, Sabrina Congedi, Maria Vittoria Chiaruttini, Elisa Pistollato, Marco Nardelli, Martina Biscaro, Mara Bassi, Giordana Coniglio, Eleonora Faccioli, Federico Rea, Dario Gregori, Paolo Navalesi, Annalisa Boscolo, the PADOVA ICU Group, Giulia Mormando, Chiara Schiavolin, Michele Della Paolera, Giovanna Pandolfo, Francesca Toma, Paola Zanon, Francesco Monteleone, Tommaso Antonio Giacon, Caterina Simoni, Arianna Peralta, Enrico Petranzan, Luisa Muraro, Paolo Persona, Giorgia Pacchiarini

**Affiliations:** 1 Institute of Anaesthesia and Intensive Care, Padua University Hospital, Padua, Italy; 2 Unit of Biostatistics, Epidemiology and Public Health, Department of Cardiac, Thoracic and Vascular Sciences, University of Padua, Padua, Italy; 3 Present Address: Department of Medicine (DIMED), Section of Anaesthesiology and Intensive Care, University of Padua, Padua, Italy; 4 Department of Cardiac, Thoracic, Vascular Sciences and Public Health, University of Padua, Padova, Italy

**Keywords:** Lung transplant, Transplantation, ECMO, Extracorporeal membrane oxygenation, CPB, Cardiopulmonary bypass

## Abstract

**Background:**

In the last decades, veno-arterial extracorporeal membrane oxygenation (V-A ECMO) has been gaining in popularity for intraoperative support during lung transplant (LT), being advocated for routinely use also in uncomplicated cases. Compared to off-pump strategy and, secondarily, to traditional cardiopulmonary bypass (CPB), V-A ECMO seems to offer a better hemodynamic stability and oxygenation, while data regarding blood product transfusions, postoperative recovery, and mortality remain unclear. This systematic review and network meta-analysis aims to evaluate the comparative efficacy and safety of V-A ECMO and CPB as compared to OffPump strategy during LT.

**Methods:**

A comprehensive literature search was conducted across multiple databases (PubMed Embase, Cochrane, Scopus) and was updated in February 2024. A Bayesian network meta-analysis (NMA), with a fixed-effect approach, was performed to compare outcomes, such as intraoperative needing of blood products, invasive mechanical ventilation (IMV) duration, intensive care unit (ICU) length of stay (LOS), surgical duration, needing of postoperative ECMO, and mortality, across different supports (i.e., intraoperative V-A (default (d) or rescue (r)) ECMO, CPB, or OffPump).

**Findings:**

Twenty-seven observational studies (6113 patients) were included. As compared to OffPump surgery, V-A ECMOd, V-A ECMOr, and CPB recorded a higher consumption of all blood products, longer IMV durations, prolonged ICU LOS, surgical duration, and higher mortalities. Comparing different extracorporeal supports, V-A ECMOd and, secondarily, V-A ECMOr overperformed CPB in nearly all above mentioned outcomes, except for RBC transfusions. The lowest rate of postoperative ECMO was recorded after OffPump surgery, while no differences were found comparing different extracorporeal supports. Finally, older age, male gender, and body mass index ≥ 25 kg/m^2^ negatively impacted on RBC transfusions, ICU LOS, surgical duration, need of postoperative ECMO, and mortality, regardless of the intraoperative extracorporeal support investigated.

**Interpretation:**

This comparative network meta-analysis highlights that OffPump overperformed ECMO and CPB in all outcomes of interest, while, comparing different extracorporeal supports, V-A ECMOd and, secondarily, V-A ECMOr overperformed CPB in nearly all above mentioned outcomes, except for RBC transfusions. Older age, male gender, and higher BMI negatively affect several outcomes across different intraoperative strategies, regardless of the intraoperative extracorporeal support investigated. Future prospective studies are necessary to optimize and standardize the intraoperative management of LT.

**Supplementary Information:**

The online version contains supplementary material available at 10.1186/s44158-024-00214-x.

## Background

Lung transplant (LT) is the definitive life-saving option for the treatment of selected patients with end-stage pulmonary disease. Although in the last decades several efforts have been made to improve short- and long-term outcomes, as a complex surgery on fragile patients, LT is burdened by high postoperative morbidity and mortality, with an estimated 5-year survival rate around 60%, lower than for all other solid organ transplants [1, 2]. Aiming at optimizing intraoperative management and maintaining hemodynamic and respiratory stability, extracorporeal life support has been increasingly applied during LT with evolving strategies [3]. While initially off-pump surgery was the traditional choice for LT and cardiopulmonary bypass (CPB) was reserved for intraoperative mechanical support only in high-risk cases, more recently veno-arterial extracorporeal membrane oxygenation (V-A ECMO) has been gaining in popularity, being applied routinely also in uncomplicated patients [4]. Actually, since the study by Hoetzenecker et al. that in a retrospective cohort of 582 bilateral LT demonstrated lower primary graft dysfunction (PGD) rate and greater survival in patients intraoperatively supported by preemptive V-A ECMO compared to those transplanted without ECMO [5], other works confirmed the beneficial effects of the routine use of default ECMO for LT compared to off-pump surgery with rescue mechanical support [3, 5–10]. Despite these promising results, the intraoperative use of extracorporeal life support for LT remains a matter of debate with no universally accepted indications and high practice variability among referral centers [3, 5–10]. Indeed, while extracorporeal mechanical support guarantees intraoperative lung protective ventilation, hemodynamic stability, and controlled graft reperfusion, minimizing the stress to the patient and grafts, it also carries the risks associated with cannulation, heparinization, and inflammatory response [3, 11–13].

Therefore, we designed the present systematic review and meta-analysis of randomized controlled trials (RCT) and observational studies, aiming at assessing among adult patients undergoing LT (P), whether the intraoperative mechanical support with CBP or V-A ECMO (I), compared to off-pump technique (C), results in different clinical outcomes (i.e., intraoperative transfusion requirements, duration of postoperative invasive mechanical ventilation (IMV), intensive care unit (ICU) length of stay (LOS), surgical duration, rate of postoperative prolongation of ECMO support, and mortality) (O).

## Materials and methods

This review was written according to the PRISMA Extension Statement for Reporting of Systematic Reviews Incorporating Network Meta-analyses of Health Care Interventions [14, 15] and according to a predefined protocol registered in PROSPERO (CRD42023421857) on May 7, 2023.

### Data sources and searches

A comprehensive search was conducted in PubMed (through Medline), Embase, Cochrane (through Ovid), and Scopus from their inception and was updated in February 2024. Supplementary Table 1 (Table S1) provides the search strategies for the four databases.

Two reviewers (PT, SN) independently screened the titles and abstracts to assess potential eligibility. Any entry identified by either reviewer advanced to the full-text eligibility review. Pretested eligibility forms were used for the full-text review, which was also conducted in duplicate. Any disagreements were resolved by a third adjudicator (BA or OH) through consensus.

### Study selection

The review focused on RCTs and observational studies that included adult patients aged 18 years or older undergoing LT and compared the effects of intraoperative V-A ECMO, whether used prophylactically (default ECMO, ECMOd) or as a rescue support in case of complications (rescue ECMO, ECMOr), and CPB versus off-pump procedures (comparator) on intraoperative and postoperative outcomes.

The main outcomes assessed were intraoperative red blood cell (RBC) transfusion (units), fresh frozen plasma (FFP) transfusion (units), platelet (PLT) transfusion (units), postoperative IMV duration (days), ICU LOS (days); then, surgical duration (hours), rate of postoperative ECMO support, and mortality (within the first 90 days after ICU admission).

### Data extraction

Data extraction was conducted by two reviewers (DCA, CS), with any disagreements resolved by an expert reviewer (BA or OH). For each eligible study, the following data were extracted: number of patients, sex, mean age, preoperative body mass index (BMI), end-stage lung disease, and all details concerning the outcomes of interest. We also collected means, standard deviations (SD), confidence intervals (CI), and significance levels for continuous data, and proportions for dichotomous data. If data were missing, a request was sent by email to the corresponding author of the study. If no response was received after the initial request, a second request was sent 1 week later. A third and final request was sent 1 week after the second one.

### Quality and certainty of evidence assessment

Two authors (DCA, CS) independently assessed the quality of the included study using the Risk Of Bias in Non-randomized Studies of Interventions (ROBINS-I) tool [16, 17], because no RTCs were included. The options for an overall RoB judgment are as follows: (i) low risk of bias, indicating the study is similar to a well-executed randomized trial; (ii) moderate risk of bias, meaning the study offers solid evidence for a non-randomized study but does not match the quality of a well-executed randomized trial; (iii) serious risk of bias, where the study has notable issues; (iv) critical risk of bias, suggesting the study is too flawed to provide useful evidence and should be excluded from any synthesis; and (v) no information available to assess the risk of bias [16]. The risk of bias plots were prepared using the *robvis* tool [18, 19].

To evaluate the credibility of our NMA results, we employed the Confidence in Network Meta-Analysis (CINeMA) tool [20]. This evaluation encompasses six critical domains: within-study bias addresses the risk of bias within the included studies; reporting bias examines the completeness and appropriateness of eligible study inclusion; indirectness assesses the relevance of the included studies to the research question; imprecision is determined by the width of CIs around the estimates; heterogeneity examines the variability in results among the contributing studies; and incoherence evaluates the consistency and transitivity assumptions. We conducted this assessment using the CINeMA web application, categorizing concerns within the evidence base as major, some, no concerns, or undetected concerns [20].

### Data synthesis and analysis

For each outcome, the following different interventions have been compared through a Bayesian network meta-analysis (NMA) with a fixed-effect approach [21]: intraoperative V-A ECMOd, ECMOr, CPB support, and off-pump surgery.

Poster distribution of the interventions effects was estimated via Markov Chain Monte Carlo (MCMC) simulations (5 chains with 50,000 iterations each, burn-in for the initial 5000 and thinning interval of 1). The analysis was conducted using the “rnmamod” package in R (version 4.3.2) (rnmamod: Bayesian Network Meta-Analysis with Missing Participants (r-project.org)).

Additionally, we explored the influence of potential mediators on the estimated interventions such as age, gender, and BMI fitting network meta-regression (NMR) models.

Further, as sensitivity analysis, we conducted subgroup analyses stratifying the included studies by risk of bias (moderate and serious), publication year (< 2010, 2010–2019, > 2020), and geographic region (USA, Europe, other).

For each outcome were reported net plots visualizing the evidence network where each node represents a different intervention, and the lines between nodes indicate direct comparisons available from the included studies. The thickness of each edge correlates with the number of trials investigating the corresponding comparison, unless specified otherwise. The Surface Under the Cumulative Ranking (SUCRA) statistics were used to rank the interventions from best to worst for each outcome based on their cumulative probabilities of being ranked at each possible position in each simulation [22].

For each pair of compared interventions, we present the summarized effect measures (EM) such as the mean difference (MD) for continuous outcomes and odds ratios (OR) for binary outcomes, along with their 95% credible intervals. These effect measures provide a quantified estimate of the difference in outcomes between two interventions, helping to guide clinical decision-making. As mediation analysis results, we present the posterior median and 95% credible interval from NMR of the MD or OR for each comparison, setting the off-pump intervention as a reference.

For model diagnostics, we checked for the convergence of the MCMC algorithm in the EM estimation and for the consistency between direct and indirect NMA estimations for those outcomes that presented at least one indirect comparison: intraoperative FFP, intraoperative PLT, postoperative ECMO, and late mortality.

Finally, the deviance information criterion (DIC) was used to compare the NMA model with the NMR model. If the difference in DIC exceeds 5, the network meta-regression model is preferred; if the difference in DIC is less than − 5, the network meta-analysis model is preferred; otherwise, models are considered equivalent [19].

## Results

### Descriptive characteristics and risk of bias of the included studies

**Fig. 1:**
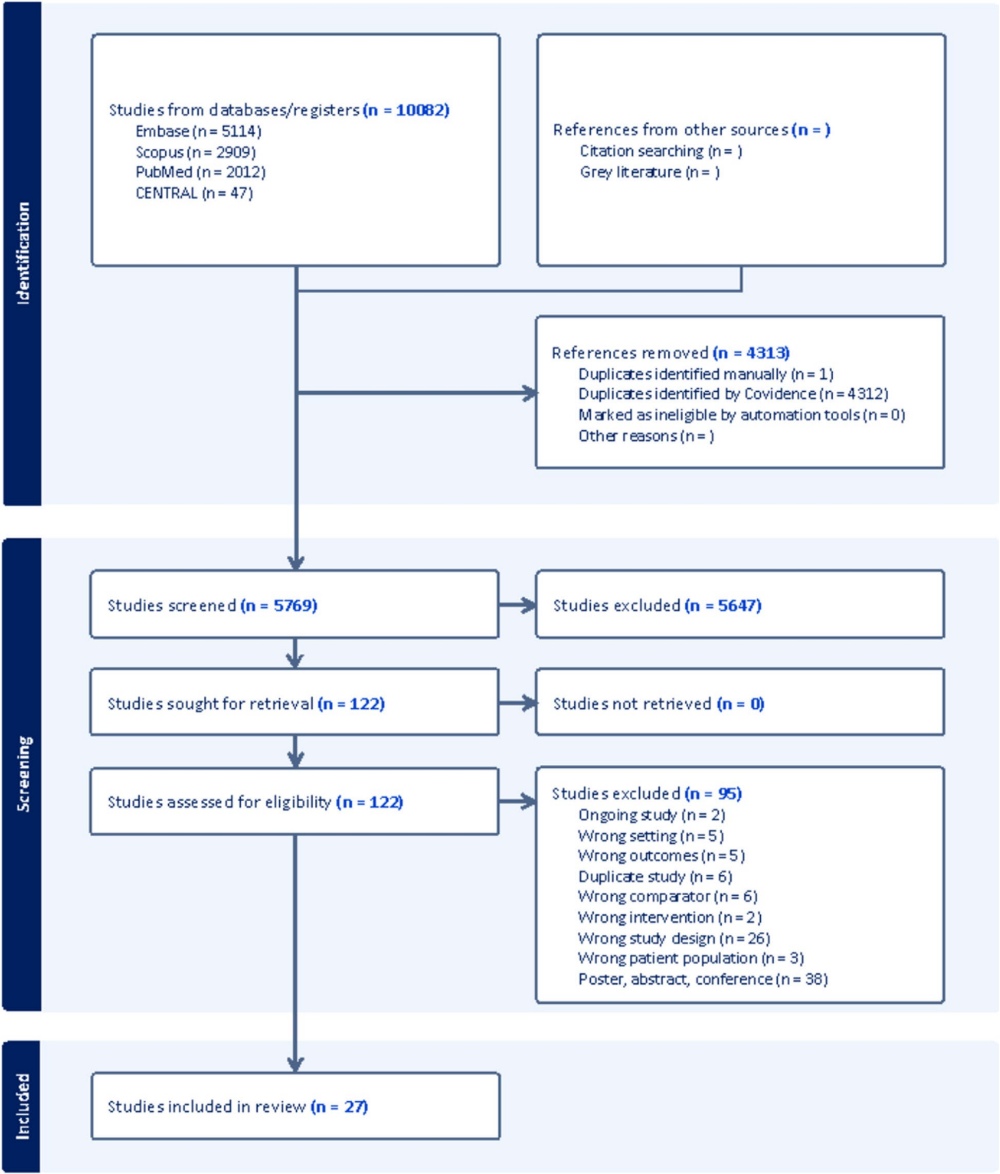
PRISMA flowchart

The search yielded a total of 10,082 results. After identifying and removing 4313 duplicates, 5769 studies remained for the title and abstract screening phase. This process resulted in 122 articles being selected for full-text assessment, of which 27 studies were ultimately included in the review, encompassing a total of 6113 patients available for analysis (Fig. 1). The characteristics of the included studies are overviewed in Table 1, while the individual contribution of the studies to each outcome is summarized in Table S2.

**Table 1:** Study characteristics

First author	Year	Country	Type of study	Inclusion criteria	Exclusion criteria	Principal outcome	Overall population (*N*)	Age (mean)	M (*n*, %)
Aigner	2007	Austria	Retrospective observational study	Patients performing LTx	Not declared	To summarize experience with ECMO in the field of LT	306 (ECMOd: 130; ECMOr: 112; off-pump: 149)	45.63	174 56.8%
Bermudez	2014	USA	Cohort study	Patients performing double LTx with intraoperatively ECLS	Not declared	To compare the early outcomes of LTx using CPB or VA-ECMO	645 (ECMOd:49; CPB: 222; off-pump: 374)	56.96	372 57.67%
Biscotti	2014	USA	Cohort study	Patients performing double LTx with ECLS	Not declared	To compare outcomes and operative parameters VA-ECMO versus CPB in LTx patients	102 (ECMOd: 47; CPB: 55)	48.69	52 50%
Bittner	2007	Germany	Cohort study	Patients performing double LTx	Multiorgan transplantation;	To report the experiences of replacing CPB with VA-ECMO support in LTx	15 (ECMOd: 8; CPB: 7)	NA	NA
Calabrese	2022	Italy	Retrospective observational study	Patients performing double LTx	Volumetric reductions in the graft; re- or multiorgan transplantation; ex vivo perfusion; bridge with ECMO to LTx	To assess ischemia in lung tissue from LTx patients receiving intraoperative VA-ECMO support and non-ECMO support	51 (ECMOd: 13; off-pump: 38)	52	17 33.3%
Chacon-Alberty	2022	USA	Cohort study	Patients performing LTx	Missing biomarker values; multiorgan transplantation;	To compare the postreperfusion circulatory cytokine patterns associated with LTx performed off-pump or with the use of CPB or VA-ECMO	59 (ECMOd:15; off-pump:26)	49.17	38 64.4%
Chan	2023	USA	Cohort study	Patients performing LTx using (i) no ECLS, (ii) VA-ECMO, and (iii) CPB	LTx on VV-ECMO, bridge with ECMO, single or re-LTx, multiorgan transplantation, or cardiac procedures	To compare outcomes of intraoperative VA-ECMO and CPB during BLTx with a propensity analysis	557 (ECMOd:150; CPB: 197; off-pump: 210)	59	320 57%
Cosgun	2017	Swiss	Cohort study	Patients performing LTx	Not declared	To investigate the outcome and risk factors on survival in recipients undergoing LTx with intraoperative ECMO support	291 (ECMOd: 134; off-pump: 157)	46.85	157 53.9%
Coster	2023	USA	Cohort study	Patients performing double LTx	Re- or multiorgan transplantation; intraoperative veno-venous ECMO, ex vivo lung perfusion	To investigate clinical outcomes of LTx in association with biomarkers of endothelial injury in conjunction with various ECLS strategies	55 (ECMOd: 22; CPB: 20; off-pump:13)	32	25 45.4%
Dalibon	2006	France	Cohort study	Patients performing double LTx	Not declared	To report the experience of planned and unplanned use of CPB for LTx	140 (CPB: 23; off-pump: 117)	41	92 65.7%
Dell’Amore	2020	Italy	Cohort study	Patients receiving double LTx for pulmonary hypertension	Absence of pulmonary hypertension	To review the results of ECLS strategy during and after BLTx for pulmonary artery hypertension	38 (ECMOd: 21; CPB: 17)	39	16 42.1%
Erkılınç	2023	Turkey	Cohort study	Patients performing double LT	ECMO as a bridge to LTx	To review experience with patients who underwent LTx with or without ECMO	48 (ECMOd: 29; off-pump: 19)	50.28	34 70.8%
Fessler	2020	France	Cohort study	Patients performing double LTx	ECMO bridge, multiorgan transplantation, re-transplantation, CPB	To assess prognoses of patients undergoing unplanned-intraoperative VA-ECMO	300 (ECMOd: 14; ECMOr: 77; off-pump: 209)	50.58	165 55%
Gammie	1998	USA	Cohort study	Patients performing double LTx	ECLS before LTx; Eisenmenger’s syndrome; over-sized double-lung graft	To assess the effect of CPB on allograft function and recipient survival in DLTx	94 (CPB: 37; off-pump:57)	NA	NA
Halpern	2022	USA	Cohort study	Patients with no or mild pulmonary hypertension performing isolated BLTx with planned off-pump or VA-ECMO	Multiorgan or single LT, moderate or severe PH, concomitant cardiac surgery, intraoperative support strategies other than off-pump or VA ECMO	To compare rates of textbook outcome between BOLTs performed with planned VA ECMO or off-pump support	237 (ECMOd:68; off-pump: 169)	61	129 54.4%
Hlozek	1997	USA	Cohort study	Single or double LTx performed with CPB support or with off-pump method	Not declared	To clarify the effect of CPB on LTx recipients	70 (CPB: 30; off-pump: 40)	NA	NA
Hoechter	2015	Germany	Cohort study	Patients performing double LTx with intraoperatively ECLS	Bridge-to-transplant ECMO therapy	To analyze transfusion requirements, coagulation parameters, and outcome parameters LTx recipients comparing CPB and ECMO	47 (ECMOd: 26; CPB: 21)	NA	NA
Hoetzenecker	2018	Austria	Cohort study	Patients performing double LTx	Single-LTx, re-transplantations, heart–LTx, bridged to transplantation	To review the results of ECMO use in LTx	582 (ECMOd: 466; off-pump: 116)	45.5	147 25.2%
Ius	2012	Germany	Cohort study	Patients performing double LTx with intraoperatively ECLS	Patients performing LTx before start of the study	To compare the postoperative course and outcomes of LTx patients treated using VA-ECMO or CPB	92 (ECMOd: 46; CPB: 46)	42.69	48 52.17%
Ius	2020	Germany	Cohort study	Patients undergoing isolated LTx with or without ECMO support	CPB use	To present the experience using intraoperative ECMO in isolated LTx evaluating its impact on long-term graft function and survival	1137 (ECMOd: 311; off-pump: 826)	51.9	593 52.15%
Loor	2022	USA, Europe	Cohort study	Patients performing double LTx	Multiorgan transplantation	To clarify the relationship between the use of ECLS during LTx and severe PGD	852 (ECMOd: 273; CPB: 157; off-pump: 422)	54.29	472 55.39%
Machuca	2015	Canada	Cohort study	Patients performing double LTx with intraoperatively ECLS	Bridge with ECLS, concomitant cardiac procedure, multiorgan transplant, colonization with *Burkholderia cenocepacia*, emergency ECLS	To compare the outcomes of intraoperative ECMO versus CPB in LTx	99 (ECMOd: 33; CPB: 66)	43.73	49 49.5%
Pettenuzzo	2018	Italy	Cohort study	Patients performing double LTx	Bridge to LTx with ECMO and/or preoperative MV	To evaluate the association of the intraoperative use of ECMO for LTx with blood products transfusion, short-term and mid-term postoperative complications	52 (ECMOd: 15; off-pump: 37)	50.73	36 69.23%
Ruszel	2021	Poland	Cohort study	Patients performing LTx	Not declared	To analyze the survival rates and frequency of complications in LTx according use of intraoperative ECMO versus CPB	63 (ECMOd: 14; CPB: 8; off-pump:37)	47.23	33 52.38%
Scaravilli	2020	Italy	Cohort study	CF patients who underwent LTx	Single LTx; re-transplantation; missing medical records	To find risk factors at the time of enlistment associated with the intraoperative use of ECLS and to compare the outcomes of CF patients treated with ECLS during LTx or not	70 (ECMOd:28; off-pump: 42)	28.2	36 51.4%
Szeto	2022	USA	Cohort study	COPD patients who underwent to double LTx	Not declared	To determine whether CPB has deleterious effects on lung function or clinical outcome	50 (CPB: 14; off-pump: 36)	50.99	31 62%
Zhao	2022	China	Cohort study	Patients performing LTx	Not declared	To review the clinical outcomes and complications of LTx recipients who received ECMO support both intra- and postoperatively in a single center	86 (ECMOd: 32; off-pump: 54)	63	22 25.6%

Abbreviations: *LTx*, lung transplant; *ECMOd*, ECMO default; *VA-ECMO*, veno-arterial ECMO; *VV-ECMO*, veno-venous ECMO; *CPB*, cardiopulmonary bypass; *ECLS*, extracorporeal life support; *MV*, mechanical ventilation; *PGD*, primary graft dysfunction; *CF*, cystic fibrosis; *COPD*, chronic obstructive pulmonary disease

**Fig. 2:**
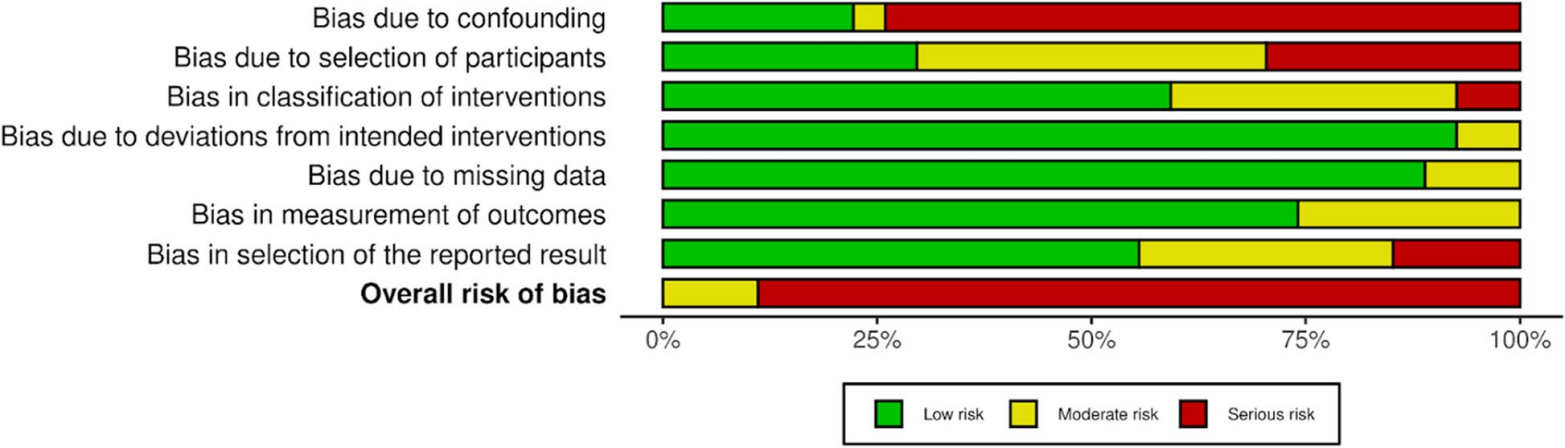
Risk of bias

Figure 2 and Tables S3 and S4 report the analysis of the risk of bias. Overall, 24 (89%) studies were rated at serious risk [5, 9, 23–45], while 3 (11%) were rated at moderate risk [46–48].

### Effects of intervention

**Fig. 3:**
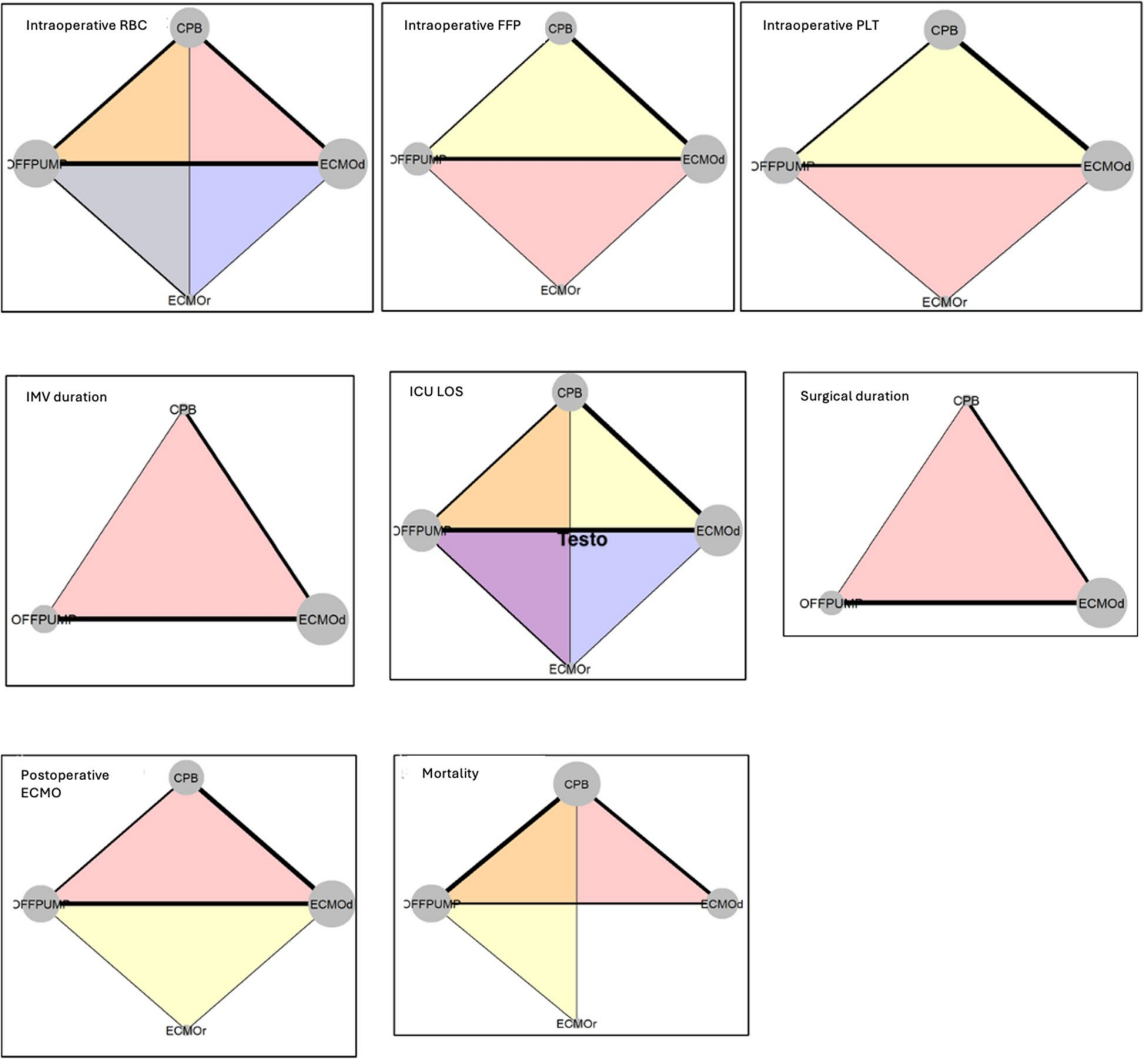
Network plots of comparative outcomes for OffPump, rescue ECMO, default ECMO, and CPB. Abbreviations: ECMOd, default ECMO; ECMOr, rescue ECMO; ECMO, extracorporeal membrane oxygenation; CPB, cardiopulmonary bypass

Figure 3 provides the net plot of the network for each outcome, while Tables 2 and S5 report the estimated overall effect measures for each outcome.

**Table 2:**
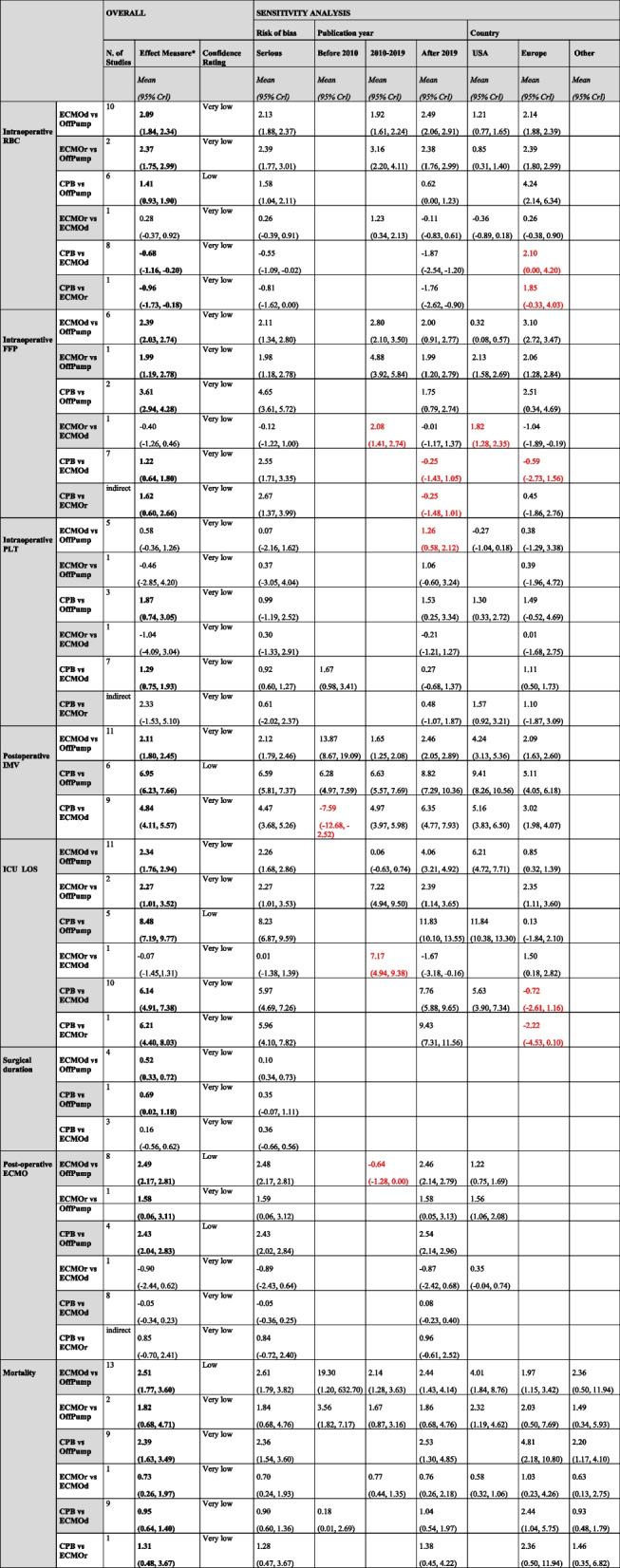
Estimated overall effect measures and sensitivity analysis (risk of bias, publication year, country). For each estimate is reported the mean effect measure and the 95% credible interval. For each estimate is reported the mean and the 95% credible interval (CrI)

^*^Data reported in bold are statistically significant. Data reported in red are different from the original findings reported in bold (*)

Abbreviations: *CrI*, credible interval; *ECMOd*, extracorporeal membrane oxygenation, decannulated; *ECMOr*, extracorporeal membrane oxygenation, recannulated; *CPB*, cardiopulmonary bypass; *OffPump*, off-pump coronary artery bypass; *IMV*, invasive mechanical ventilation; *RBC*, red blood cells; *FFP*, fresh frozen plasma; *PLT*, platelets; *USA*, United States of America; *LOS*, length of stay

#### RBC, FFP, and PLT transfusions

Compared to off-pump strategy, all intraoperative extracorporeal supports were associated to a greater need of RBC transfusions (ECMOd: mean 2.09 units, 95% CrI 1.84–2.34; ECMOr: mean 2.37 units, 95% CrI 1.75–2.99; and CPB: mean 1.41 units, 95% CrI 0.93–1.90, respectively). In addition, CPB overperformed both ECMOd and ECMOr in terms of RBC transfusions (mean − 0.68 units, 95% CrI − 1.16, − 0.20 and − 0.96 units, 95% CrI − 1.73, − 0.18, respectively).

ECMOd, ECMOr, and CPB needed more FFP transfusions (mean 2.39 units, 95% CrI 2.03–2.74; mean 1.99 units, 95% CrI 1.19–2.78; and mean 3.61 units, 95% CrI 2.94–4.28, respectively). However, CPB required more FFPs than ECMOd and ECMOr (mean 1.22 units, 95% CrI 0.64–1.80 and mean 1.62 units, 95% CrI 0.60–2.66, respectively).

Concerning PLT transfusions, only CPB needed more PLTs as compared to off-pump surgery (mean 1.87 units, 95% CrI 0.74–3.05) and compared to ECMOd (mean 1.29 units, 95% CrI 0.75–1.93).

#### IMV duration

Compared to OffPump strategy, ECMOd and CPB required longer postoperative IMV (mean 2.11 days, 95% CrI 1.80–2.45 and mean 6.95 days, 95% CrI 6.23–7.66, respectively), and CPB performed worse than ECMOd (mean 4.84 days, 95% CrI 4.11–5.57).

#### ICU LOS

Compared to OffPump strategy, ECMOd, ECMOr, and CPB were characterized by more prolonged ICU LOS (mean 2.34 days, 95% CrI 1.76–2.94; mean 2.27 days, 95% CrI 1.01–3.52; and mean 8.48 days, 95% CrI 7.19–9.77, respectively), and CPB performed worse as compared to ECMOd or ECMOr (mean 6.14 days, 95% CrI 4.91–7.38 and mean 6.21 days, 95% CrI 4.40–8.03, respectively).

#### Surgical duration

Surgical duration was barely longer during ECMOd (mean 0.52 h, 95% CrI 0.33–0.72) and CPB (mean 0.69 h, 95% CrI 0.02–1.18), as compared to OffPump surgery.

#### Postoperative ECMO support

A greater incidence of postoperative ECMO support was assessed considering all extracorporeal supports in comparison to OffPump strategy, with no differences between different extracorporeal supports.

#### Mortality

Mortality was greater during ECMOd (mean 2.51, 95% CrI 1.77–3.60), ECMOr (mean 1.82, 95% CrI 0.68–4.71), and CPB (mean 2.39, 95% CrI 1.63–3.49), compared to OffPump surgery. While, among extracorporeal supports, ECMOd was associated with the lowest mortality (mean 0.73, 95% CrI 0.26–1.97, compared to ECMOr, and mean 0.95, 95% CrI 0.64–1.40 compared to CPB).

### Sensitivity analysis

The results of the sensitivity analysis according to RoB, publication year, and country are shown in Table 2.

Most results concerning the need of blood products were confirmed except for:

i) RBC transfusions, more frequently requested during CPB as compared to ECMOd (mean 2.10 units, 95% CrI 0.00–4.20) or ECMOr (mean 1.85 units, 95% CrI − 0.33–4.03), considering only studies published in Europe.
ii) FFP transfusions, because ECMOd overperformed ECMOr either considering publications realized between 2010 and 2019 (2.08, 95% CrI 1.41–2.74) or those papers published in America (1.82, 95% CrI 1.28–2.35). In addition, CPB decreased the need of FFP transfusions, as compared to ECMOd and ECMOr, considering only publications released after 2019 and from Europe.

Considering postoperative IMV, ECMOd was associated with a shorter duration of IMV after LT compared to CPB (mean − 7.59 days, 95% CrI − 12.68, − 2.52) considering the oldest publications.

When examining ICU LOS, ECMOr performed worse as compared to ECMOd (mean 7.17 days, 95% CrI 4.94–9.38) considering publications released between 2010 and 2019. Conversely, CPB performed better as compared to ECMOd (mean − 0.72 days, 95% CrI − 2.61–1.16) and to ECMOr (mean − 2.22 days, 95% CrI − 4.53–0.10), considering papers from Europe.

Finally, ECMOd overperformed OffPump surgery in terms of postoperative ECMOs in the case of papers published between 2010 and 2019 (mean − 0.64, 95% CrI − 1.28–0.00).

### Mediation analysis

The overall effect estimates for each outcome were recalculated based on the studies with available mediation variables (i.e., age, gender, BMI) (Table 3), and the values reported under different conditions tell us how much the estimated mean effect of the mediation changes when we consider different levels of the mediating variables.

**Table 3:** Mediation analysis considering age (< 50 and ≥ 50 years), gender (female, male), and body mass index (BMI < 25 and BMI ≥ 25). For each mediator is reported the network meta-analysis (NMA) and the network meta-regression (NMR) with the 95% credible intervals (CrI)

	Versus OffPump	Age			Gender			Body mass index (BMI)		
		Overall	Age < 50 years	Age ≥ 50 years	Overall	Female	Male	Overall	BMI < 25 kg/m^2^	BMI ≥ 25 kg/m^2^
		Mean (95% CrI) NMA	Mean **(**95% CrI) NMR	Mean (95% CrI) NMR	Mean (95% CrI) NMA	Mean (95% CrI) NMR	Mean (95% CrI) NMR	Mean (95% CrI) NMA	Mean (95% CrI) NMR	Mean (95% CrI) NMR
**Intraoperative RBC**	**ECMOd**	2.08 (1.83, 2.34)*	2.34 (1.93, 2.75)*	1.96 (1.3, 2.62)*	2.05 (1.8, 2.3)*	2.12 (1.82, 2.43)*	2.04 (1.45, 2.63)*	2.03 (1.79, 2.28)*	1.82 (1.53, 2.12)*	2.26 (1.71, 2.81)*
	**ECMOr**	2.35 (1.73, 2.96)*	2.32 (1.7, 2.93)*	0.08 (− 4.28, 4.43)	2.35 (1.73, 2.97)*	2.36 (1.74, 2.97)*	2.59 (− 0.39, 5.57)	2.38 (1.76, 3.01)*	1.95 (− 0.96, 4.87)	2.36 (− 1.72, 6.44)
	**CPB**	1.37 (0.86, 1.89)*	4.74 (3.5, 5.98)*	0.62 (− 1.22, 2.45)	1.40 (0.89, 1.91)*	1.07 (0.53, 1.61)*	4.25 (2.54, 5.95)*	1.29 (0.73, 1.84)*	1.01 (− 1.85, 3.88)	1.43 (− 2.6, 5.45)
**Intraoperative FFP**	**ECMOd**	2.15 (1.67, 2.6)*	2.70 (2.05, 3.35)*	2.13 (1.08, 3.17)*	2.33 (1.93, 2.77)*	2.17 (1.56, 2.78)*	2.45 (1.51, 3.38)*	2.09 (1.59, 2.56)*	1.14 (0.18, 2.1)*	2.69 (1.35, 4.04)*
	**ECMOr**	2.01 (1.21, 2.82)*	2.04 (1.25, 2.83)*	− 0.21 (− 4.41, 3.99)	2.02 (1.23, 2.82)*	2.01 (1.21, 2.8)*	2.16 (− 0.07, 4.39)	2.01 (1.2, 2.81)*	0.45 (− 2.55, 3.45)	2.02 (− 2.15, 6.18)
	**CPB**	3.38 (2.56, 4.21)*	5.74 (4.64, 6.83)*	1.88 (0.12, 3.63)*	3.43 (2.63, 4.2)*	3.38 (2.45, 4.31)*	3.45 (1.95, 4.95)*	3.80 (2.92, 4.72)*	2.45 (− 0.66, 5.56)	4.02 (− 0.27, 8.3)
**Postoperative IMV**	**ECMOd**	2.13 (1.82, 2.45)*	1.68 (1.31, 2.05)*	2.98 (2.21, 3.75)*	2.28 (1.95, 2.65)*	2.23 (1.83, 2.63)*	2.42 (1.63, 3.2)*	2.55 (2.16, 3.05)*	2.19 (1.69, 2.69)*	2.99 (2.1, 3.88)*
	**CPB**	7.43 (6.59, 8.28)*	7.17 (5.67, 8.67)*	7.64 (5.4, 9.88)*	7.02 (6.31, 7.75)*	7.12 (6.27, 7.97)*	6.96 (5.5, 8.43)*	8.79 (7.42, 10.15)*	8.08 (4.94, 11.23)*	8.91 (4.66, 13.15)*
**ICU LOS**	**ECMOd**	2.21 (1.63, 2.8)*	− 0.44 (− 1.14, 0.25)	5.68 (4.34, 7.02)*	2.47 (1.83, 3.14)*	2.36 (1.49, 3.23)*	2.64 (1.09, 4.18)*	3.61 (2.73, 4.48)*	2.30 (0.75, 3.85)*	4.12 (1.72, 6.52)*
	**ECMOr**	2.27 (1.02, 3.53)*	2.10 (0.85, 3.36)*	7.97 (4.98, 10.96)*	2.27 (1.02, 3.53)*	2.27 (1.03, 3.52)*	2.84 (− 0.13, 5.82)	2.34 (1.09, 3.6)*	0.56 (− 2.89, 4.02)	2.35 (− 2.37, 7.07)
	**CPB**	8.09 (6.75, 9.44)*	4.29 (2.23, 6.35)*	9.89 (6.91, 12.86)*	8.57 (7.27, 9.88)*	8.38 (6.97, 9.79)*	9.15 (6.53, 11.76)*	10.98 (9.5, 12.46)*	9.35 (5.87, 12.84)*	11.16 (6.39, 15.93)*
**Surgical duration**	**ECMOd**	0.52 (0.34, 0.72)*	1.18 (0.65, 1.71)*	− 0.95 (− 1.78, − 0.13)*	0.53 (0.34, 0.72)*	0.37 (0.12, 0.61)*	3.59 (2.64, 4.53)*	0.34 (0, 0.56)*	0.75 (0.51, 0.99)*	− 0.98 (− 1.45, − 0.5)*
	**CPB**	0.68 (0.02, 1.18)*	1.96 (− 0.94, 4.86)	− 0.15 (− 4.24, 3.95)	0.56 (− 0.04, 1.15)	0.39 (− 0.21, 1)	3.88 (2.45, 5.3)*	0.49 (− 0.05, 1.03)	1.61 (− 1.24, 4.46)	− 0.13 (− 4.16, 3.91)
**Postoperative ECMO**	**ECMOd**	12.27 (8.95, 17.04)*	3.70 (0.29, 46.61)	12.46 (0.35, 447.07)	12.21 (8.92, 16.98)*	7.51 (4.72, 11.94)*	17.96 (8.09, 39.9)*	11.93 (8.71, 16.59)*	1.40 (0.65, 3.01)	20.73 (6.55, 65.66)*
	**ECMOr**	4.89 (1.06, 22.61)*	3.95 (0.78, 19.99)	19.07 (0.41, 896.43)	4.93 (1.06, 23.16)*	4.16 (0.91, 18.94)	9.85 (0.97, 100)	4.90 (1.07, 22.82)*	0.41 (0.02, 7.05)	6.20 (0.14, 269.13)
	**CPB**	10.94 (7.35, 16.43)*	1.79 (0.11, 29.99)	11.42 (0.21, 613.7)	11.56 (7.82, 17.23)*	7.74 (4.82, 12.44)*	17.00 (6.06, 47.66)*	11.60 (7.76, 17.44)*	1.29 (0.12, 14.17)	18.87 (0.63, 566.85)
**Mortality**	**ECMOd**	2.61 (1.83, 3.74)*	2.02 (1.19, 3.4)*	3.04 (1.25, 7.39)*	2.44 (1.7, 3.52)*	2.53 (1.58, 4.04)*	2.27 (1, 5.17)*	2.45 (1.52, 4.03)*	1.27 (0.48, 3.37)	3.02 (0.69, 13.31)
	**ECMOr**	1.81 (0.67, 4.65)	1.70 (0.65, 4.44)	3.13 (0.32, 31)	1.82 (0.68, 4.69)	1.98 (0.68, 5.83)	1.60 (0.35, 7.32)	2.08 (0.51, 7.88)	0.88 (0.03, 25.79)	2.16 (0.02, 208.82)
	**CPB**	2.13 (1.43, 3.17)*	1.21 (0.59, 2.49)	2.72 (0.89, 8.29)	2.28 (1.54, 3.38)*	2.43 (1.56, 3.8)*	1.94 (0.8, 4.68)	1.70 (0.91, 3.2)	0.79 (0.04, 15.64)	2.01 (0.03, 138.93)

Abbreviations: *CrI*, credible interval; *ECMOd*, extracorporeal membrane oxygenation, decannulated; *ECMOr*, extracorporeal membrane oxygenation, recannulated; *CPB*, cardiopulmonary bypass; *OffPump*, off-pump coronary artery bypass; *IMV*, invasive mechanical ventilation; *RBC*, red blood cells; *FFP*, fresh frozen plasma; *PLT*, platelets; *USA*, United States of America; *LOS*, length of stay. *: significant results

Considering age, the most relevant effects were recorded in ICU LOS, remarkably longer among patients aged above 50 or older, and in surgical duration, shorter in older patients, irrespective of the intraoperative support. All other findings were confirmed.

With regard to gender, male patients required more blood transfusions, experienced longer surgical durations, and a greater need of postoperative ECMOs compared to female patients and across different intraoperative strategies.

Finally, patients with a BMI above 25 kg/m^2^ experienced worse outcomes in terms of ICU LOS, needing postoperative ECMO, and mortality, regardless of intraoperative strategy.

### SUCRA

As shown in Table 4, off-pump surgery consistently ranked highest, with a posterior mean of 1.00 (95% CI 1.00–1.00), recording the most favorable outcomes in terms of lower need of RBC and FFP transfusions, shorter postoperative IMV, and ICU LOS.

**Table 4:** Comparative effectiveness of surgical procedures: SUCRA values for each intervention for each outcome

		Posterior mean	2.5%	97.5%
**Intraoperative RBC**	OffPump	1.00	1.00	1.00
	ECMO default	0.27	0.00	0.33
	ECMO rescue	0.07	0.00	0.33
	CPB	0.66	0.67	0.67
**Intraoperative FFP**	OffPump	1.00	1.00	1.00
	ECMO default	0.39	0.33	0.67
	ECMO rescue	0.61	0.33	0.67
	CPB	0.00	0.00	0.00
**Intraoperative PLT**	OffPump	0.66	0.33	1.00
	ECMO default	0.53	0.16	0.67
	ECMO rescue	0.74	0.00	1.00
	CPB	0.07	0.00	0.33
**Postoperative IMV**	OffPump	1.00	1.00	1.00
	ECMO default	0.5	0.5	0.5
	CPB	0.00	0.00	0.00
**ICU LOS**	OffPump	1.00	1.00	1.00
	ECMO default	0.49	0.33	0.67
	ECMO rescue	0.51	0.33	0.67
	CPB	0.00	0.00	0.00
**Surgical duration**	OffPump	0.98	0.5	1.00
	ECMO default	0.29	0.00	0.50
	CPB	0.23	0.00	1.00
**Postoperative ECMO**	OffPump	0.99	1.00	1.00
	ECMO default	0.16	0.00	0.67
	ECMO rescue	0.59	0.00	0.67
	CPB	0.26	0.00	0.67
**Mortality**	OffPump	0.96	0.67	1.00
	ECMO default	0.22	0.00	0.67
	ECMO rescue	0.51	0.00	1.00
	CPB	0.30	0.00	0.67

For each intervention is reported the posterior mean and the 95% confidence interval (CI)

Abbreviations: *ECMOd*, extracorporeal membrane oxygenation, decannulated; *ECMOr*, extracorporeal membrane oxygenation, recannulated; *CPB*, cardiopulmonary bypass; *OffPump*, off-pump coronary artery bypass; *IMV*, invasive mechanical ventilation; *RBC*, red blood cells; *FFP*, fresh frozen plasma; *PLT*, platelets; *LOS*, length of stay

Moreover, OffPump demonstrated favorable benefits also in terms of lower rates of postoperative ECMOs (posterior mean of 0.99, 95% CI 1.00–1.00) and mortality (posterior mean of 0.96, 95% CI 0.67–1.00).

### Quality of the evidence

We assessed the quality of the evidence across various outcomes using the CINeMA tool, which resulted in a very low confidence rating for most comparisons (Table 2). The key factors that reduced the quality of evidence were the high within-study bias. This bias stemmed mainly from methodological limitations (i.e., inadequate randomization, lack of blinding, and incomplete data handling). In many cases, reporting bias was assessed as low risk, but other domains such as indirectness, imprecision, and heterogeneity frequently presented concerns. For most comparisons, the evidence was downgraded by at least two levels due to major concerns in multiple domains.

The lack of adequate studies across some comparisons further compounded the issue, limiting our ability to test for publication bias or to evaluate consistency between direct and indirect evidence. The frequent presence of heterogeneity and incoherence, especially in comparisons with very low confidence, indicated that the effects might be less reliable.

## Discussion

The present systematic review and meta-analysis, based on 27 observational studies enrolling 6113 patients, provide comprehensive insights into perioperative and postoperative outcomes in LT recipients undergoing different intraoperative support (i.e., ECMOd, ECMOr, or CPB), as compared to OffPump strategy. The preliminary findings of this analysis suggest that OffPump overperformed ECMO and CPB in all outcomes of interest, while, comparing different extracorporeal supports, V-A ECMOd and, secondarily, V-A ECMOr overperformed CPB in nearly all above mentioned outcomes, except for RBC transfusions. In fact, CPB is associated with prolonged recovery and weaning from IMV, greater intraoperative needing of FFP and PLTs, while not of RBCs.

To the best of our knowledge, the present systematic review and meta-analysis is the first investigation, focused on LT, exclusively aiming to evaluate the comparative efficacy and safety of V-A ECMO and CPB, as compared to off-pump strategy, using a well-designed Bayesian qualitative analysis.

Although the preliminary findings confirmed data of previous studies [5, 9, 23–48], a few notable deviations emerged after the sensitivity analysis. Indeed, our analysis revealed that RBCs and FFP requirements were influenced by geographical and temporal variations. The geographical shifts could be explained, at least in part, by different clinical practices or perioperative protocols between European and American hospitals, impacting on transfusion thresholds. Conversely, changes in coagulopathy management and the progressive replacing of CPB with intraoperative VA-ECMO at the different institutions may have influenced the temporal paradigm. Indeed, the most recent reports demonstrated lower blood product transfusion rates in the ECMO group relative to the CPB one [31, 37, 41, 46]. However, no similar data are available on previous analysis and no comparison is possible.

In keeping with previous studies, on perioperative outcomes of mechanical support strategy in LT patients [32, 34, 48], our updated meta-analysis confirms that off-pump and ECMOd groups experienced a shorter IMV duration and ICU LOS, as compared to CPB patients. Notably, our results, corroborating previous findings, highlight the significant impact of the variables such as age and BMI on ICU LOS, the rate of postoperative ECMOs, and mortality. In fact, older patients and those recipients with a BMI above 25 kg/m^2^ experienced longer ICU LOS. Even though historically, overweight LT recipients have been linked with poor post-surgical outcomes and BMI was incorporated as a component of the lung allocation score [49, 50], in a recent retrospective cohort of 108 bilateral LT adult recipients, a linear relation was reported among the BMI and ICU LOS [51]. Moreover, the relevant impact of the age on ICU LOS probably reflects the general observation that older patients tend to have more complex recoveries, regardless of the intraoperative mechanical support provided to LT recipients. Similarly, the older age seems to negatively impact also on surgical duration, probably due to more difficult cannulation, a higher risk of cardiovascular disease, and hemodynamic instability [52].

Finally, consistent with the result of the largest and most recent studies on this topic [7, 10], investigating the mortality rate in LT patients according to extracorporeal support strategy, our meta-analysis confirms that OffPump strategy overperforms all extracorporeal supports, although we observed a great heterogeneity among enrolled populations (i.e., cystic fibrosis, severe pulmonary hypertension), and concerning clinical indications for intraoperative extracorporeal support. In fact, in the last decades, several authors reported promising results also in favor of ECMOd, and not only in the case of off-pump [7, 10], and although our analysis suggests that off-pump procedures appear superior to ECMO and CPB in the various outcomes considered, studies supporting ECMOd show positive results not only in the most critically ill recipients but also in recipients with mixed profiles. Moreover, the use of intraoperative support could limit and prevent the onset of severe PGD (an outcome not included in our analysis due to the high heterogeneity of the extracted data, which rendered it unanalyzable). Therefore, the development of prospective, ideally randomized and controlled studies is warranted to assess the impact of ECMO use on PGD prevention and survival, with standardized timing for data collection. Although our analysis suggests that off-pump procedures appear superior to ECMO and CPB in the various outcomes considered, promising initial results have also been reported with the use of ECMOd. Studies supporting ECMOd show positive results not only in the most critically ill patients but also in mixed cohorts, particularly in efforts to limit or prevent the onset of severe PGD (an outcome not included in our analysis due to the high heterogeneity of the extracted data, which rendered it unanalyzable)^3,5,7,11,,34,35,36^. Therefore, the development of prospective, ideally randomized and controlled studies is warranted to assess the impact of ECMO use on PGD prevention and survival, with standardized timing for data collection.

### Limitations

Some limitations need to be declared. Firstly, a significant portion of included studies exhibit a serious risk of bias across various domains, particularly in confounding and outcome measurement. This raises concerns about the reliability of the study findings. Moreover, the considerable heterogeneity among studies in terms of design, patient populations, and clinical practices may introduce substantial variability, making it challenging to draw definitive conclusions from the pooled data. Furthermore, the limited availability of data on key variables, such as patient characteristics and procedural details, may hinder the accuracy and comprehensiveness of the analysis. Additionally, not all potential confounding factors are adequately accounted for in the analyses, which could lead to biased effect estimates and undermine the validity of the results. Finally, while mediation analysis provides insights into the mechanisms underlying treatment effects, the inability to fully control for all mediating variables may introduce uncertainty and limit the interpretability of the findings.

## Conclusions

This comparative network meta-analysis highlights that OffPump overperformed ECMO and CPB in all outcomes of interest, while, comparing different extracorporeal supports, V-A ECMOd and, secondarily, V-A ECMOr overperformed CPB in nearly all outcomes of interest (i.e., such as intraoperative needing of FFP and PLTs, IMV duration, ICU LOS, surgical duration, needing of postoperative ECMO, and mortality), except for RBC transfusions. Older age, male gender, and higher BMI negatively affect several outcomes across different intraoperative strategies, regardless of the intraoperative extracorporeal support investigated. Future prospective studies are necessary to optimize and standardize the intraoperative management of LT. Future prospective studies are necessary to confirm these findings and optimize the intraoperative management of LT.

## Supplementary Information

Supplementary Material 1.

## Data Availability

No datasets were generated or analysed during the current study.

## Abbreviations

V-A ECMO: Veno-arterial extracorporeal membrane oxygenation
LT: Lung transplant
CPB: Cardiopulmonary bypass
NMA: Network meta-analysis
IMV: Invasive mechanical ventilation
ICU: Intensive care unit
LOS: Length of stay
d: Default
r: Rescue
PGD: Primary graft dysfunction
RBC: Red blood cell
FFP: Fresh frozen plasma
PLT: Platelet
BMI: Body mass index
SD: Standard deviations
CI: Confidence intervals
ROBINS-I: Risk Of Bias in Non-randomized Studies of Interventions
CINeMA: Confidence in Network Meta-Analysis
MCMC: Markov Chain Monte Carlo
NMR: Network meta-regression
SUCRA: Surface Under the Cumulative Ranking
EM: Effect measures
MD: Mean difference
OR: Odds ratios
DIC: Deviance information criterion
