# Supplementary material for: Intraoperative extracorporeal support for lung transplant: a systematic review and network meta-analysis
Source: J Anesth Analg Crit Care. 2024 Dec 18;4:81. doi: 10.1186/s44158-024-00214-x (PMC11658095; doi:10.1186/s44158-024-00214-x)
Supplement: Supplementary file 1 — Supplementary Material 1. [file 44158_2024_214_MOESM1_ESM.docx]

**Supplementary material**

**Table S1. Search strategies for PubMed, Scopus, Embase and Cochrane. Last updated was done on February 2, 2024.**

**Table S2. Contribution of individual studies to each outcome.**

**Table S3. Risk-of-bias judgements within each bias domain.**

**Table S4. Level of concern for each of the six domains and the summarizing judgment for each comparison.**

**Table S5. Summary of findings table.**

**Table S1. Search strategies for PubMed, Scopus, Embase and Cochrane. Last updated was done on February 2, 2024.**

| PubMed | ("extracorporeal membrane oxygenation"[MeSH Terms] OR "ecmo"[Title/Abstract] OR "extracorporeal circulation"[MeSH Terms] OR "extracorporeal circulation"[Title/Abstract] OR "circulation extracorporeal"[Title/Abstract] OR (("blood circulation"[MeSH Terms] OR ("blood"[All Fields] AND "Circulation"[All Fields]) OR "blood circulation"[All Fields] OR "Circulation"[All Fields] OR "Circulations"[All Fields] OR "circulate"[All Fields] OR "circulated"[All Fields] OR "circulates"[All Fields] OR "circulating"[All Fields]) AND "Extracorporeal"[Title/Abstract]) OR "extracorporeal circulations"[Title/Abstract] OR "Extracorporeal Membrane Oxygenations"[Title/Abstract] OR "membrane oxygenation extracorporeal"[Title/Abstract] OR "oxygenation extracorporeal membrane"[Title/Abstract] OR "ECMO Treatment"[Title/Abstract] OR "ECMO Treatments"[Title/Abstract] OR "treatment ecmo"[Title/Abstract] OR "ECLS Treatment"[Title/Abstract] OR "ECLS Treatments"[Title/Abstract] OR "treatment ecls"[Title/Abstract] OR "ECMO Extracorporeal Membrane Oxygenation"[Title/Abstract] OR "Extracorporeal Life Support"[Title/Abstract] OR "Extracorporeal Life Supports"[Title/Abstract] OR "life support extracorporeal"[Title/Abstract] OR "Venoarterial ECMO"[Title/Abstract] OR "ecmo venoarterial"[Title/Abstract] OR "Venoarterial Extracorporeal Membrane Oxygenation"[Title/Abstract] OR "Venovenous ECMO"[Title/Abstract] OR "ecmo venovenous"[Title/Abstract] OR "Venovenous Extracorporeal Membrane Oxygenation"[Title/Abstract] OR "cardiopulmonary bypass"[MeSH Terms] OR "cardiopulmonary bypass"[Title/Abstract] OR "heart lung bypass"[Title/Abstract] OR "bypass heart lung"[Title/Abstract] OR "heart lung bypass"[Title/Abstract] OR "bypass cardiopulmonary"[Title/Abstract] OR "bypasses cardiopulmonary"[Title/Abstract] OR "Cardiopulmonary Bypasses"[Title/Abstract]) AND ("lung transplantation"[MeSH Terms] OR "lung transplantation"[Text Word] OR "grafting lung"[Title/Abstract] OR "Lung Grafting"[Title/Abstract] OR "transplantation lung"[Title/Abstract] OR "Lung Transplantations"[Title/Abstract] OR "transplantations lung"[Title/Abstract]) |
| --- | --- |
| Scopus | TITLE-ABS-KEY ( "extracorporeal membrane oxygenation" OR ecmo OR "Extracorporeal Membrane Oxygenations" OR "Membrane Oxygenation, Extracorporeal" OR "Oxygenation, Extracorporeal Membrane" OR "ECMO Treatment" OR "ECMO Treatments" OR "Treatment, ECMO" OR "ECLS Treatment" OR "ECLS Treatments" OR "Treatment, ECLS" OR "ECMO Extracorporeal Membrane Oxygenation" OR "Extracorporeal Life Support" OR "Extracorporeal Life Supports" OR "Life Support, Extracorporeal" OR "Venoarterial ECMO" OR "ECMO, Venoarterial" OR "Venoarterial ECMOs" OR "Venoarterial Extracorporeal Membrane Oxygenation" OR "Venovenous ECMO" OR "ECMO, Venovenous" OR "Venovenous ECMOs" OR "Venovenous Extracorporeal Membrane Oxygenation" OR "cardiopulmonary bypass" OR "Heart-Lung Bypass" OR "Bypass, Heart-Lung" OR "Bypasses, Heart-Lung" OR "Heart Lung Bypass" OR "Heart-Lung Bypasses" OR "Bypass, Cardiopulmonary" OR "Bypasses, Cardiopulmonary" OR "Cardiopulmonary Bypasses" OR "VA&#8722;ECLS" OR "VA&#8722;ECMO" OR "vaECLS" OR "vaECMO" OR "veno arterial extracorporeal oxygenation" ) AND TITLE-ABS-KEY ( "lung transplantation" OR "Grafting, Lung" OR "Graftings, Lung" OR "Lung Grafting" OR "Lung Graftings" OR "Transplantation, Lung" OR "Lung Transplantations" OR "Transplantations, Lung" ) |
| Embase | extracorporeal circulation.tw. or extracorporeal circulation/ or extracorporeal membrane oxygenation.tw. or exp extracorporeal oxygenation/ or exp extracorporeal membrane oxygenation device/ or ecmo.tw. or "Extracorporeal Membrane Oxygenations".tw. or "Membrane Oxygenation, Extracorporeal".tw. or "Oxygenation, Extracorporeal Membrane".tw. or "ECMO Treatment".tw. or "ECMO Treatments".tw. or "Treatment, ECMO".tw. or "ECLS Treatment".tw. or "ECLS Treatments".tw. or "Treatment, ECLS".tw. or "ECMO Extracorporeal Membrane Oxygenation".tw. or "Extracorporeal Life Support".tw. or "Extracorporeal Life Supports".tw. or "Life Support, Extracorporeal".tw. or "Venoarterial ECMO".tw. or "ECMO, Venoarterial".tw. or "Venoarterial ECMOs".tw. or "Venoarterial Extracorporeal Membrane Oxygenation".tw. or "Venovenous ECMO".tw. or "ECMO, Venovenous".tw. or "Venovenous ECMOs".tw. or "Venovenous Extracorporeal Membrane Oxygenation".tw. or "VA−ECLS".tw. or "VA−ECMO".tw. or "vaECLS".tw. or "vaECMO".tw. or "veno arterial extracorporeal oxygenation".tw. or veno-venous ECMO/ or veno-arterial ECMO/ or cardiopulmunary bypass.tw. or exp cardiopulmonary bypass/ or "Heart-Lung Bypass".tw. or "Bypass, Heart-Lung".tw. or "Bypasses, Heart-Lung".tw. or "Heart Lung Bypass".tw. or "Heart-Lung Bypasses".tw. or "Bypass, Cardiopulmonary".tw. or "Bypasses, Cardiopulmonary".tw. or "Cardiopulmonary Bypasses".tw. |
|  | lung transplantation.tw. or exp lung transplantation/ or "Grafting, Lung".tw. or "Graftings, Lung".tw. or "Lung Grafting".tw. or "Lung Graftings".tw. or "Transplantation, Lung".tw. or "Lung Transplantations".tw. or "Transplantations, Lung".tw. |
| Cochrane |  |

**Table S2. Contribution of Individual Studies to each outcome.**

| **Outcome** | **Studies** | **N participants** |
| --- | --- | --- |
| Intraoperative RBC | Bermudez 2014, Biscotti 2014, Chacon;Alberty 2022, Chan 2023, Coster 2023, Dalibon 2006, Fessler 2020, Halpern 2022, Hoechter 2015, Hoetzenecker 2018, Ius 2012, Ius 2020, Machuca 2015, Pettenuzzo 2018, Ruszel 2021, Scaravilli 2020, Szeto 2002, Zhao 2022 | 4001 |
| Intraoperative FFP | Bermudez 2014, Biscotti 2014, Chacon-Alberty 2022, Coster 2023, Fessler 2020, Halpern 2022, Hoechter 2015, Hoetzenecker 2018, Ius 2012, Ius 2020, Machuca 2015 | 2983 |
| Intraoperative PLT | Bermudez 2014, Biscotti 2014, Chacon-Alberty 2022, Coster 2023, Fessler 2020, Halpern 2022, Hoechter 2015, Ius 2012, Ius 2020, Machuca 2015, Szeto 2002 | 2451 |
| Post-operative IMV | Aigner 2007, Bermudez 2014, Bittner 2007, Chan 2023, Cosgun 2017, Coster 2023, Dalibon 2006, Dell'Amore 2020, Erkılınç 2023, Gammie 1998, Hoechter 2015, Hoetzenecker 2018, Ius 2012, Ius 2020, Machuca 2015, Pettenuzzo 2018, Scaravilli 2020, Zhao 2022 | 4338 |
| ICU LOS | Aigner 2007, Bermudez 2014, Biscotti 2014, Bittner 2007, Chacon-Alberty 2022, Chan 2023, Cosgun 2017, Coster 2023, Dell'Amore 2020, Erkılınç 2023, Fessler 2020, Hoechter 2015, Ius 2012, Ius 2020, Machuca 2015, Pettenuzzo 2018, Scaravilli 2020, Zhao 2022 | 2451 |
| Surgical duration | Bittner 2007, Chan 2023, Cosgun 2017, Hoechter 2015, Hoetzenecker 2018, Zhao 2022, Bittner 2007, Chan 2023, Cosgun 2017, Hoechter 2015, Hoetzenecker 2018, Zhao 2022 | 1580 |
| Post-operative ECMO | Bermudez 2014, Biscotti 2014, Chacon-Alberty 2022, Chan 2023, Coster 2023, Erkılınç 2023, Fessler 2020, Halpern 2022, Hoechter 2015, Ius 2020, Loor 2022, Machuca 2015 | 3766 |
| Mortality | Bermudez 2014, Biscotti 2014, Bittner 2007, Calabrese 2022, Chacon-Alberty 2022, Cosgun 2017, Coster 2023, Dalibon 2006, Dell'Amore 2020, Erkılınç 2023, Fessler 2020, Gammie 1998, Halpern 2022, Hlozek 1997, Hoechter 2015, Hoetzenecker 2018, Loor 2022, Machuca 2015, Pettenuzzo 2018, Ruszel 2021, Scaravilli 2020, Szeto 2002, Zhao 2022 | 4048 |
|  |  |  |
|  |  |  |
|  |  |  |
|  |  |  |
|  |  |  |
|  |  |  |
|  |  |  |

**Table S3. Risk-of-bias judgements within each bias domain.**
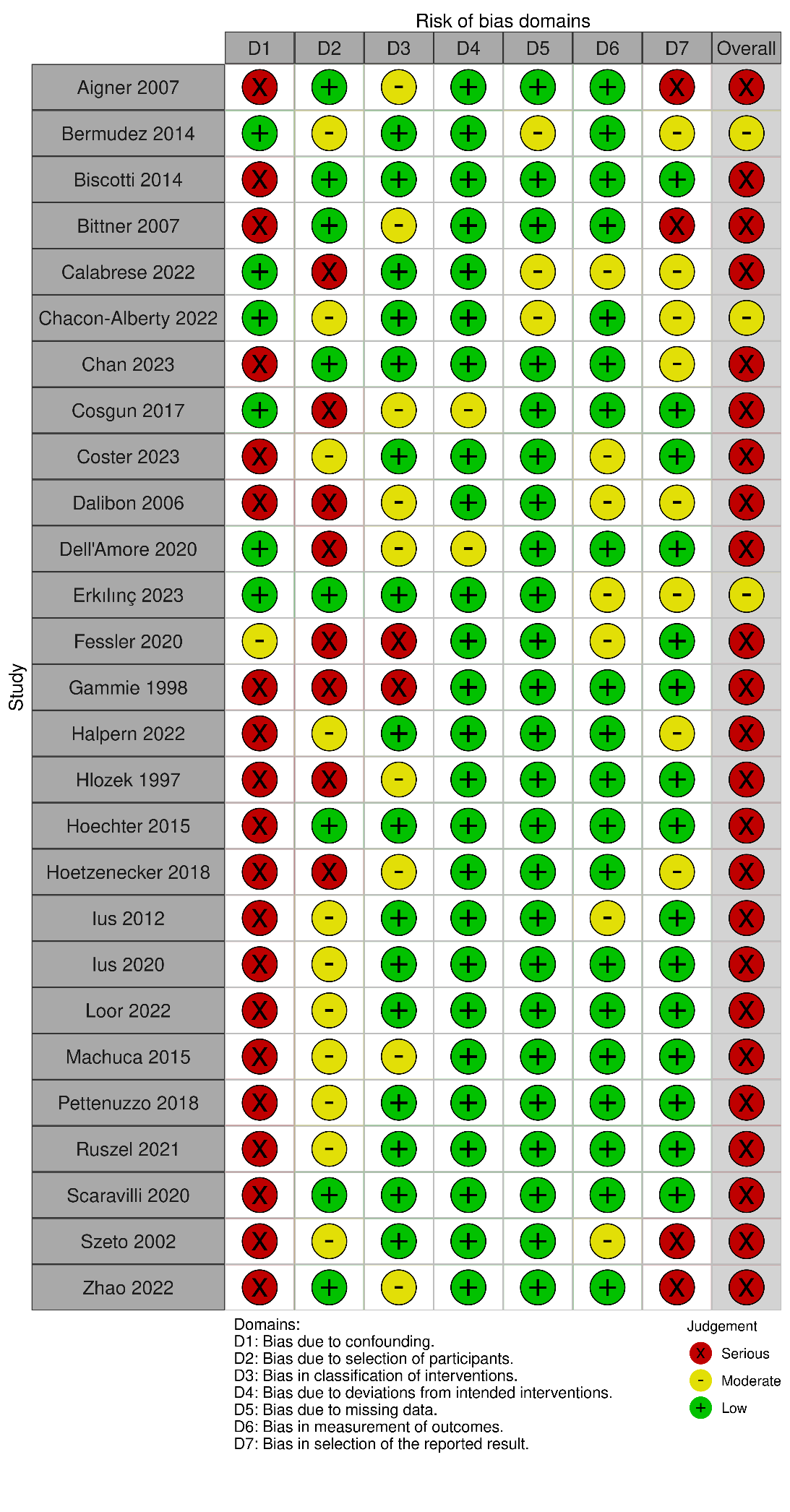


**Table S4. Level of concern for each of the six domains and the summarizing judgment for each comparison.**

|  | **Comparison** | **N. of studies** | **With-in study bias** | **Reporting bias** | **Indirectness** | **Imprecision** | **Heterogeneity** | **Incoherence** | **Confidence rating** |
| --- | --- | --- | --- | --- | --- | --- | --- | --- | --- |
| **Intra operative RBC** | ECMOd vs OffPump | 10 | Major concerns | Low risk | Major concerns | No concerns | Major concerns | Major concerns | Very low |
|  | ECMOr vs OffPump | 2 | Major concerns | Some concerns | Major concerns | No concerns | Major concerns | No concerns | Very low |
|  | CPB vs OffPump | 6 | Major concerns | Some concerns | Major concerns | No concerns | Some concerns | Some concerns | Low |
|  | ECMOr vs ECMOd | 1 | Major concerns | Some concerns | Major concerns | No concerns | Major concerns | No concerns | Very low |
|  | CPB vs ECMOd | 8 | Major concerns | Some concerns | Major concerns | No concerns | Major concerns | No concerns | Very low |
|  | CPB vs ECMOr | 1 | Major concerns | Low risk | Major concerns | Low risk | Major concerns | Major concerns | Very low |
| **Intra operative FFP** | ECMOd vs OffPump | 6 | Major concerns | Low risk | Some concerns | No concerns | Major concerns | Major concerns | Very low |
|  | ECMOr vs OffPump | 1 | Major concerns | Low risk | Some concerns | No concerns | Major concerns | No concerns | Very low |
|  | CPB vs OffPump | 2 | Major concerns | Low risk | Some concerns | No concerns | Major concerns | No concerns | Very low |
|  | ECMOr vs ECMOd | 1 | Major concerns | Low risk | Some concerns | Some concerns | Some concerns | No concerns | Very low |
|  | CPB vs ECMOd | 7 | Major concerns | Low risk | Some concerns | No concerns | Major concerns | Major concerns | Very low |
|  | CPB vs ECMOr | indirect | Major concerns | Low risk | Some concerns | No concerns | Major concerns | No concerns | Very low |
| **Intra operative PLT** | ECMOd vs OffPump | 5 | Major concerns | Some concerns | Some concerns | No concerns | Major concerns | No concerns | Very low |
|  | ECMOr vs OffPump | 1 | Major concerns | Some concerns | Some concerns | No concerns | Major concerns | No concerns | Very low |
|  | CPB vs OffPump | 3 | Major concerns | Some concerns | Some concerns | No concerns | Major concerns | Some concerns | Very low |
|  | ECMOr vs ECMOd | 1 | Major concerns | Some concerns | Some concerns | No concerns | Major concerns | No concerns | Very low |
|  | CPB vs ECMOd | 7 | Major concerns | Some concerns | Some concerns | No concerns | Major concerns | No concerns | Very low |
|  | CPB vs ECMOr | indirect | Major concerns | Some concerns | Some concerns | No concerns | Major concerns | No concerns | Very low |
| **Post-operative IMV LOS** | ECMOd vs OffPump | 11 | Major concerns | Some concerns | Some concerns | No concerns | Some concerns | No concerns | Very low |
|  | CPB vs OffPump | 6 | Major concerns | Some concerns | Some concerns | No concerns | No concerns | No concerns | Low |
|  | CPB vs ECMOd | 9 | Major concerns | Some concerns | Some concerns | No concerns | Major concerns | No concerns | Very low |
| **ICU LOS** | ECMOd vs OffPump | 11 | Major concerns | Low risk | Some concerns | No concerns | Major concerns | No concerns | Very low |
|  | ECMOr vs OffPump | 2 | Major concerns | Low risk | Some concerns | No concerns | Major concerns | Some concerns | Very low |
|  | CPB vs OffPump | 5 | Major concerns | Low risk | Some concerns | No concerns | No concerns | Some concerns | Low |
|  | ECMOr vs ECMOd | 1 | Major concerns | Low risk | Some concerns | Some concerns | Some concerns | Major concerns | Very low |
|  | CPB vs ECMOd | 10 | Major concerns | Low risk | Some concerns | No concerns | Major concerns | No concerns | Very low |
|  | CPB vs ECMOr | 1 | Major concerns | Low risk | Some concerns | No concerns | Major concerns | No concerns | Very low |
| **Surgical duration** | ECMOd vs OffPump | 4 | Major concerns | Low risk | Some concerns | No concerns | Major concerns | No concerns | Very low |
|  | CPB vs OffPump | 1 | Major concerns | Low risk | Some concerns | No concerns | Major concerns | Major concerns | Very low |
|  | CPB vs ECMOd | 3 | Major concerns | Low risk | Some concerns | No concerns | Major concerns | Major concerns | Very low |
| **Mortality** | ECMOd vs OffPump | 13 | Major concerns | Low risk | Some concerns | No concerns | No concerns | No concerns | Low |
|  | ECMOr vs OffPump | 2 | Major concerns | Some concerns | Some concerns | Major concerns | No concerns | No concerns | Very low |
|  | CPB vs OffPump | 9 | Major concerns | Low risk | Some concerns | No concerns | No concerns | Major concerns | Very low |
|  | ECMOr vs ECMOd | 1 | Major concerns | Low risk | Some concerns | Major concerns | No concerns | No concerns | Very low |
|  | CPB vs ECMOd | 9 | Major concerns | Low risk | Some concerns | Major concerns | No concerns | No concerns | Very low |
|  | CPB vs ECMOr | 1 | Major concerns | Low risk | Some concerns | Major concerns | No concerns | No concerns | Very low |

**Abbreviations**: ECMOd: Extracorporeal Membrane Oxygenation, Decannulated, ECMOr: Extracorporeal Membrane Oxygenation, Recannulated, CPB: Cardiopulmonary Bypass, OffPump: Off-Pump Coronary Artery Bypass, IMV: Invasive Mechanical Ventilation, RBC: Red Blood Cells, FFP: Fresh Frozen Plasma, PLT: Platelets; LOS: length of stay.

**Table S5. Summary of findings table.**

|  | **Comparison** | **N. of studies** | **Confidence rating** | **Mean 95% CrI** | **Interpretations of findings** |
| --- | --- | --- | --- | --- | --- |
| **OUTCOMES** | |  |  |  |  |
| **Intra operative RBC** | ECMOd vs OffPump | 10 | Very low | **2.09 (1.84, 2.34)** | Uncertain evidence of ECMOd overperforming OffPump |
|  | ECMOr vs OffPump | 2 | Very low | **2.37 (1.75, 2.99)** | Uncertain evidence of ECMOr overperforming OffPump |
|  | CPB vs OffPump | 6 | Low | **1.41 (0.93, 1.90)** | Uncertain evidence of CPB overperforming OffPump |
|  | ECMOr vs ECMOd | 1 | Very low | 0.28 (-0.37, 0.92) |  |
|  | CPB vs ECMOd | 8 | Very low | -0.68 (-1.16, -0.20) |  |
|  | CPB vs ECMOr | 1 | Very low | **-0.96 (-1.73, -0.18)** | Uncertain evidence of CPB overperforming ECMOr |
| **Intra operative FFP** | ECMOd vs OffPump | 6 | Very low | **2.39 (2.03, 2.74)** | Uncertain evidence of ECMOd overperforming OffPump |
|  | ECMOr vs OffPump | 1 | Very low | **1.99 (1.19, 2.78)** | Uncertain evidence of ECMOr overperforming OffPump |
|  | CPB vs OffPump | 2 | Very low | **3.61 (2.94, 4.28)** | Uncertain evidence of CPB overperforming OffPump |
|  | ECMOr vs ECMOd | 1 | Very low | -0.40 (-1.26, 0.46) |  |
|  | CPB vs ECMOd | 7 | Very low | **1.22 (0.64, 1.80)** | Uncertain evidence of CPB overperforming ECMOd |
|  | CPB vs ECMOr | indirect | Very low | **1.62 (0.60, 2.66)** | Uncertain evidence of CPB overperforming ECMOr |
| **Intra operative PLT** | ECMOd vs OffPump | 5 | Very low | 0.58 (-0.36, 1.26) |  |
|  | ECMOr vs OffPump | 1 | Very low | -0.46 (-2.85, 4.20) |  |
|  | CPB vs OffPump | 3 | Very low | **1.87 (0.74, 3.05)** | Uncertain evidence of CPB overperforming OffPump |
|  | ECMOr vs ECMOd | 1 | Very low | -1.04 (-4.09, 3.04) |  |
|  | CPB vs ECMOd | 7 | Very low | **1.29 (0.75, 1.93)** | Uncertain evidence of CPB overperforming ECMOd |
|  | CPB vs ECMOr | indirect | Very low | 2.33 (-1.53, 5.10) |  |
| **Post-operative IMV length** | ECMOd vs OffPump | 11 | Very low | **2.11 (1.80, 2.45)** | Uncertain evidence of ECMOd overperforming OffPump |
|  | CPB vs OffPump | 6 | Low | **6.95 (6.23, 7.66)** | Moderate evidence of CPB overperforming OffPump |
|  | CPB vs ECMOd | 9 | Very low | **4.84 (4.11, 5.57)** | Uncertain evidence of CPB overperforming ECMOd |
| **ICU LOS** | ECMOd vs OffPump | 11 | Very low | **2.34 (1.76, 2.94)** | Uncertain evidence of ECMOd overperforming OffPump |
|  | ECMOr vs OffPump | 2 | Very low | **2.27 (1.01, 3.52)** | Uncertain evidence of ECMOr overperforming OffPump |
|  | CPB vs OffPump | 5 | Low | **8.48 (7.19, 9.77)** | Moderate evidence of CPB overperforming OffPump |
|  | ECMOr vs ECMOd | 1 | Very low | -0,07 (-1,45,1.31) |  |
|  | CPB vs ECMOd | 10 | Very low | **6.14 (4.91, 7.38)** | Uncertain evidence of CPB overperforming ECMOd |
|  | CPB vs ECMOr | 1 | Very low | **6.21 (4.40, 8.03)** | Uncertain evidence of CPB overperforming ECMOr |
| **Surgical duration** | ECMOd vs OffPump | 4 | Very low | **0.52 (0.33, 0.72)** | Uncertain evidence of ECMOd overperforming OffPump |
|  | CPB vs OffPump | 1 | Very low | **0.69 (0.02, 1.18)** | Uncertain evidence of CPB overperforming OffPump |
|  | CPB vs ECMOd | 3 | Very low | 0.16 (-0.56, 0.62) |  |
| **Post-operative ECMO** | ECMOd vs OffPump | 8 | Low | **2.49 (2.17, 2.81)** | Moderate evidence of ECMOd overperforming OffPump |
|  | ECMOr vs OffPump | 1 | Very low | **1.58 (0.06, 3.11)** | Uncertain evidence of ECMOr overperforming OffPump |
|  | CPB vs OffPump | 4 | Low | **2.43 (2.04, 2.83)** | Moderate evidence of CPB overperforming OffPump |
|  | ECMOr vs ECMOd | 1 | Very low | -0.90 (-2.44, 0.62) |  |
|  | CPB vs ECMOd | 8 | Very low | -0.05 (-0.34, 0.23) |  |
|  | CPB vs ECMOr | indirect | Very low | 0.85 (-0.70, 2.41) |  |
| **Mortality** | ECMOd vs OffPump | 13 | Low | **2.51 (1.77, 3.60)** | Moderate evidence of ECMOd overperforming OffPump |
|  | ECMOr vs OffPump | 2 | Very low | **1.82 (0.68, 4.71)** | Uncertain evidence of ECMOr overperforming OffPump |
|  | CPB vs OffPump | 9 | Very low | **2.39 (1.63, 3.49)** | Uncertain evidence of CPB overperforming OffPump |
|  | ECMOr vs ECMOd | 1 | Very low | **0.73 (0.26, 1.97)** | Uncertain evidence of ECMOr overperforming ECMOd |
|  | CPB vs ECMOd | 9 | Very low | **0.95 (0.64, 1.40)** | Uncertain evidence of CPB overperforming ECMOd |
|  | CPB vs ECMOr | 1 | Very low | **1.31 (0.48, 3.67)** | Uncertain evidence of CPB overperforming ECMOr |
|  |  |  |  |  |  |

To note, findings underlined in orange recorded a moderate grade of evidence; while those compared underlined in gray derived from an analysis of less than 5 studies.

**Abbreviations**: ECMOd: Extracorporeal Membrane Oxygenation, Decannulated, ECMOr: Extracorporeal Membrane Oxygenation, Recannulated, CPB: Cardiopulmonary Bypass, OffPump: Off-Pump Coronary Artery Bypass, IMV: Invasive Mechanical Ventilation, RBC: Red Blood Cells, FFP: Fresh Frozen Plasma, PLT: Platelets; LOS: length of stay.
